# Preparation and Characterization of Aminoglycoside-Loaded Chitosan/Tripolyphosphate/Alginate Microspheres against *E. coli*

**DOI:** 10.3390/polym13193326

**Published:** 2021-09-28

**Authors:** Estefanía Tiburcio, Eduardo García-Junceda, Leoncio Garrido, Alfonso Fernández-Mayoralas, Julia Revuelta, Agatha Bastida

**Affiliations:** 1 BioGlycoChem Group, Institute of General Organic Chemistry (CSIC), Juan de la Cierva 3, 28006 Madrid, Spain; tiburcio@uv.es (E.T.); eduardo.junceda@csic.es (E.G.-J.); alfonso.mayoralas@csic.es (A.F.-M.); 2 Nanohybrids and Interactive Polymers Group, Institute of Polymer Science and Technology (ICTP-CSIC), CSIC, Juan de la Cierva 3, 28006 Madrid, Spain; lgarrido@cetef.csic.es

**Keywords:** antibiotic delivery, chitosan particles, polysaccharide carriers, release kinetics, antibiotic activity

## Abstract

Although aminoglycosides are one of the common classes of antibiotics that have been widely used for treating infections caused by pathogenic bacteria, the evolution of bacterial resistance mechanisms and their inherent toxicity have diminished their applicability. Biocompatible carrier systems can help sustain and control the delivery of antibacterial compounds while reducing the chances of antibacterial resistance or accumulation in unwanted tissues. In this study, novel chitosan gel beads were synthesized by a double ionic co-crosslinking mechanism. Tripolyphosphate and alginate, a polysaccharide obtained from marine brown algae, were employed as ionic cross-linkers to prepare the chitosan-based networks of gel beads. The in vitro release of streptomycin and kanamycin A was bimodal; an initial burst release was observed followed by a diffusion mediated sustained release, based on a Fickian diffusion mechanism. Finally, in terms of antibacterial properties, the particles resulted in growth inhibition of Gram-negative (*E. coli*) bacteria.

## 1. Introduction

Microbial infections have become a major problem in public health, and it has been anticipated that if no prior action is taken, these infections could lead to 10 million people dying every year by 2050 [1]. This threat is majorly due, on the one hand, to the increased emergence of drug-resistant microbes, which poses the serious risk of reversing previous medical progress in this field and bringing back many bacterial infections from the past. On the other hand, the development of new antibiotics has declined since 1980, which could be due to difficulties in clinical development and scientific, regulatory, and economic issues. In fact, in the past 20 years, only two new antibiotic classes have been approved by the FDA [2]. Based on these facts, the current studies are aimed towards the discovery of novel techniques to overcome these relevant changes, “re-employing” conventional antibacterial drugs [3,4]. 

Drug delivery vehicles have been widely employed to encapsulate and deliver conventional antibiotics, improving their therapeutic index while minimizing their adverse effects [5,6,7]. These have shown unprecedented advantages such as enhancement of the effectiveness of existent antibiotics, by enhancing the physicochemical properties and stability of antibiotics and prolongation of antibiotic release, in addition to the capability of targeted delivery to the site of infection as an effective antimicrobial therapy [8,9,10]. 

Microspheres are of particular interest as antibacterial drug delivery systems, representing novel strategies of targeted drug delivery systems to alleviate the increasing resistance crisis [11]. Their large specific surface area favors the interaction with target tissues allowing the direct treatment application directly to tissues, rather than delivery via bodily fluids, keeping the antibiotic effectiveness even if a lower dose is administrated. In this sense, although both synthetic and natural polymers have been employed to obtain microspheres for controlled-release drug delivery [12,13,14,15], polysaccharide-based ones in their wide diversity have constituted an important research line in recent years, due to their diverse bioactive attributes as well as other functional properties (biocompatibility, stability, etc.) [16].

**Figure 1:**
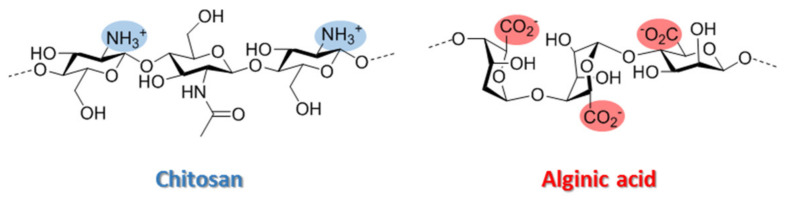
Chemical structures of chitosan and alginic acid.

Chitosan (Ch) (Figure 1) is a sustainable polysaccharide derived from renewable feedstock which has many advantages, including good biocompatibility, strong biodegradable ability, mucoadhesive properties and low toxicity [17]. Consequently, Ch-based biomaterials are versatile scaffolds for drug delivery systems development due to their unique qualities [18]. Ch has a cationic nature due to the protonation of amino groups on the polymer backbone, allowing it to interact with anions [19]. The concept of polyelectrolyte complex (PEC) formation between oppositely charged polymers has been known and used for decades in different applications, including the development of spherical carriers for drug delivery. Thus, polymer–polymer cross-linking has been described as an interesting approach to obtain superior and biocompatible Ch hydrogels and has enabled the combination of Ch with other important biopolymers that can synergize to improve encapsulation efficiency, release and bioavailability [20]. 

Alginate (Alg) (Figure 1) is a commonly employed polysaccharide to form Chit-Alg polyelectrolyte complexes (PECs). These materials have interesting properties, such as a good swelling behavior at different pHs and a better structural and mechanical stability than the isolated polymers. However, improving these properties remains a great challenge today. 

Nowadays there is the question of how to improve the stability of Ch-based polyelectrolyte complexes during swelling in different media without losing their pH-dependent swelling behavior. PECs can be reinforced through the formation of additional chemical cross-links between polyelectrolyte chains, being glutaraldehyde [21,22] the most common cross-linker used for Ch/Alg complex reinforcement. However, this cross-linking agent may be associated with toxic and other undesirable effects of these particles that can limit their application, especially in biomedicine and pharmacy. Ionotropic gelation of chitosan by means of tripolyphosphate (TPP) represents a safe efficient alternative to covalent crosslinking because of milder condition of use and, in general, higher biocompatibility of the resulting systems. This reagent is by far the most employed cross-linker to ionically reticulate chitosan due to its high net negative charges (ranging from one to five depending on pH) per monomeric unit and has successfully exploited to obtain chitosan nanoparticles and nano/micro-gels for drug delivery [23,24].

In recent years, many techniques have been employed for chitosan particles preparation, being ionic gelation and polyelectrolyte complexation the most extensively employed for drug delivery. [25,26,27]. However, to date, there are very few studies focused on evaluating the antimicrobial effect provided by drugs such as aminoglycosides (AGAs) loaded in chitosan particles cross-linked with different polymeric anionic agents [20,28,29,30]. Due to the net positive charge caused by the amino groups in biological conditions, AGAs could be candidates for affinity-based encapsulation to create an antibacterial controlled release system.

These antibiotics are a family of critically important antibiotics for the treatment of serious infections [31]. However, their clinical use has been compromised by widespread instances of resistance [32] or adverse side effects such as ototoxicity and nephrotoxicity at high antibiotic doses [33], being the preparation of new aminoglycoside formulations that allow on-demand drug delivery a plausible solution to this sticky issue. Poly (lactic acid) or poly (lactic-co-glycolic acid)-based systems have been commonly employed for AGA encapsulation [34,35,36]; however, the formation of PLGA and PLA particles requires the use of organic solvents. This is a major drawback since, among other effects, the use of organic solvents can produce relevant toxicological effects and, moreover, can reduce the scalability and practical industrial application of these systems.

The goal of this study was to employ a polyionic complexation approach using chitosan and alginate/TPP to create particles in a single step by a simple mechanism, to provide a sustainable and efficient antibacterial platform with sustained and controlled release of AGAs, and subsequently demonstrate their antibacterial activity. 

## 2. Materials and Methods

### 2.1. Materials

Chitosan (high molecular weight, batch STB06500; M_W_ = 310‒375 kDa, DD ≥ 75%) and alginic acid (from brown algae, batch SLBQ3216V; M_W_ ≈ 240 kDa, 61% of β‒(1→4)-D-mannosyluronic acid and 39% of α (1→4)-L-gulosyluronic acid residues, approximately) were purchased from Sigma-Aldrich (Sigma-Aldrich, Saint Louis, MO, USA). Tripolyphosphate (TPP) was purchased from Alfa-Aesar (Alfa Aesar, Haverhill, MA, USA). Commercial-grade reagents were used without further purification. Centrifugations were performed with an Eppendorf centrifuge 5804R instrument (Eppendorf AG, Hamburg, Germany) at 4 °C. The bacterial strain utilized in this study (*E. coli* BL21) was obtained from CECT (Spanish Type Culture Collection, Valencia, Spain). 

### 2.2. Preparation of Beads

**Figure 2:**
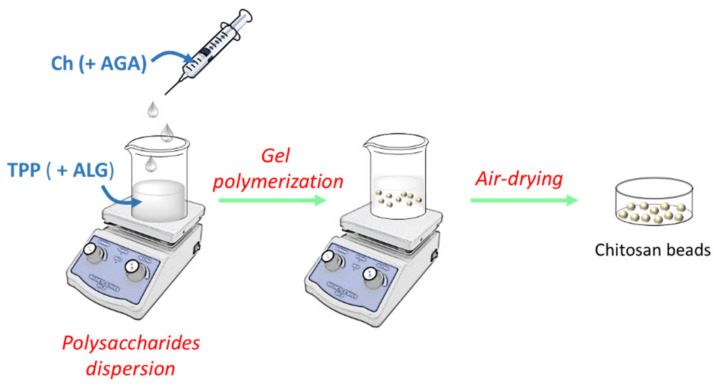
General flow chart of the preparation of chitosan-based beads.

The AGA-loaded hydrogel beads were prepared using modifications of a previously described method [37] in three steps: polysaccharides dispersion (step 1), gel polymerization (step 2) and air-drying (step 3), according with the procedure presented in Figure 2. 

#### 2.2.1. Step 1: Polysaccharides’ Dispersion

A chitosan dispersion (1.7% (*w*/*v*) was prepared by adding the required amount of polymer in a 2% (*v*/*v*) solution of acetic acid under stirring. The system was left to stand for 24 h at room temperature for complete hydration of the polymer and removal of the bubbles, and subsequently, the pH of the solution was increased from 3 to 5 employing a 1.0 M NaOH solution, while the solution was mixed using vortex agitation for 2–3 min. For AGA inclusion, a solution of AGA (≈50 mg) in water (100 μL) was prepared and, next, was added slowly to 2 mL of the chitosan solution. In parallel, a solution of TPP (3% *w*/*v*) and alginate (2% *w*/*v*) in water was prepared. Both solutions were stirred until homogeneity.

#### 2.2.2. Step 2: Precipitation of Beads

The chitosan/AGA solution (4 mL) was dropped by using a syringe into 25 mL of cross-linking solution stirred with 300 rpm on a magnetic stirred at room temperature. The beads were prepared by keeping them in the cross-linking solution under the static condition for 15 min at room temperature. 

#### 2.2.3. Step 3: Air-Drying

Finally, the beads were removed from the solution, were left to dry in a Petri dish for 24 h at room temperature, and then collected. 

### 2.3. Physico-Chemical Characterization

Microspheres were characterized using previously described methods [38,39]. 

#### 2.3.1. FTIR Spectroscopy

FT-IR spectra were recorded with KBr pellets on a One B Perkin-Elmer Spectrum spectrophotometer (Perkin-Elmer, Waltham, MA, USA). Fine powders of the samples were mixed with KBr powder in the ratio 1:100, and the mixture was subjected to a load of 10 ton/cm^2^ to produce a disc. Spectra were recorded in the absorbance mode in the range of 4000.0 and 400 cm^−1^ with a resolution of 4 cm^−1^. 

#### 2.3.2. Microscopic Characterization

Microscopic characterization of microspheres was done to investigate the morphology of particles by using a 2MP zoom stereo microscope (resolution 1600 × 1200, Tangxi, Chizhou, China). This microscopic evaluation was performed by using sediment after centrifugation of the microsphere suspension. The sediment was placed on object glass covered with a cover glass. Then, the observation was made using a 20× magnification lens.

#### 2.3.3. Scanning Electron Microscopy 

Shape and size of beads was analyzed by Scanning Electron Microscopy (SEM) at a bead concentration of 0.04 mg/mL using a Hitachi S-8000 (Hitachi, Tokyo, Japan) operating in transmission mode at 100 kV on dry samples.

#### 2.3.4. Degree of Swelling

The swelling rates of desiccated beads in a saline phosphate buffer solution (50 mM, pH = 7.4, 100 mM NaCl) were followed using a 2MP zoom stereo microscope (resolution 1600 × 1200, Tangxi, Chizhou, China). The degree of swelling was estimated as the ratio of the area of the projected surface of the particles [ø(d)], compared to that of freshly prepared swollen beads [ø(s)]. The degree of swelling was therefore calculated according to:

(1) $$\mathrm{Degree}\;\mathrm{of}\;\mathrm{swelling}\;\left(\%\right)=\frac{ø\left(s\right)-ø\left(d\right)}{ø\left(d\right)}\times 100$$

#### 2.3.5. Degradation

Dry microspheres with a diameter of 0.51 mm were immersed in PBS (pH = 7.4) and the area of the projected surface of the particles (ø (mm)) was measured at different times.

### 2.4. Antibiotic Activity

The inhibition of bacterial growth by Chit/TPP/Alg microspheres charged with kanamycin and streptomycin was examined measuring zones of growth inhibition by the agar disk diffusion assay [40]. Briefly, a standardized inoculum of the microorganism (*E. coli*) is swabbed onto the surface of LB-agar plate, and then, AGA in solution as control or AGA microspheres were directly added onto the plate. After 24 h of culture in static conditions at 37 °C, the inhibition zone was analyzed. The normalized width of the antimicrobial “halo” [nw (halo)] of each disk was calculated according to:

(2) $$\mathrm{nw}\;\left(\mathrm{halo}\right)=\frac{\frac{d\left(iz\right)-d}{2}}{d}$$

where *d(iz)* is the outer diameter of the inhibition zone and *d* the disk diameter. 

### 2.5. Drug Loading and Loading Efficiency

The drug loading of the beads was estimated according to the agar disk-diffusion test. Briefly, 25 mg of microspheres suspended in 200 μL of saline PBS (pH = 7.4) were placed in an Amicon Ultra 10K centrifugal filter and subject to centrifugation at 10,000× *g* for 10 min at room temperature. The filtrate was collected and loaded on foams placed later in contact with a pre-seeded of *E. coli* and the “halo” was measured after 24 h of culture in static conditions at 37 °C. Calibration curves were prepared by determination of antimicrobial “halo” of known antibiotics concentrations (1 μg/mL, 10 μg/mL and 100 μg/mL) in PBS. The drug loading and loading efficiency were calculated from the following equation, respectively:

(3) $$\mathrm{Drug}\;\mathrm{loading}\;\left(\%,\;w/w\right)=\frac{\mathrm{Mass}\;\mathrm{of}\;\mathrm{drug}\;\mathrm{in}\;\mathrm{microspheres}}{\mathrm{Mass}\;\mathrm{of}\;\mathrm{microspheres}}\;x\;100$$

(4) $$\mathrm{Drug}\;\mathrm{loading}\;\mathrm{efficiency}\;\left(\%,\;w/w\right)=\frac{\mathrm{Drug}\;\mathrm{loading}}{\mathrm{Theorical}\;\mathrm{drug}\;\mathrm{loading}\;}\;x\;100$$

### 2.6. Cumulative Drug Release

The release of drug from the microspheres was estimated in PBS (pH = 7.4) at 37 °C by agar disk-diffusion test. A volume of 200 microliters of the buffer solution was sampled periodically at predetermined intervals and was replaced with the same volume of fresh PBS (pH 7.4). The extracted aliquots were loaded on foams placed later in contact with a pre-seeded of *E. coli* BL21 agar plates according with the procedure described previously (Section 2.5). The amount of released antibiotic was calculated as cumulative percent release.

### 2.7. Drug Release Kinetics

Different models (zero order, first order, Higuchi square root of time, and Hixson-Crowell models) were evaluated to understand the kinetics of drug release. [41]. The best fit was evaluated by calculating the correlation coefficient, according with the following equations, respectively:

(5) $$F=K_{O}\;t$$

(6) $$F=1-e^{-K_{1}t}$$

(7) F=KHt12

(8) $$\sqrt[{3}]{F}=K_{S}t$$

where F is the drug release fraction at time t (F = Mt/M∞) in which Mt is the drug-released percentage at time t and M∞ is the total drug-release percentage. Time has been normalized as t/t∞ where t∞ is the total experiment time. 

Additionally, to understand the release mechanism, the release data were fitted to the Korsmeyer–Peppas model according to the following equation:

(9) $$F={\mathrm{Kt}}^{n}$$

The exponent “*n*” is known as “diffusional exponent” and is related to the release mechanism, being obtained from the plot of ln (F) versus ln (t). When *n* = 0.5, it is assumed that a Fickian diffusion occurs (diffusion-controlled transport), while a *n* = 0.5–1.0 is associated to an anomalous or non-Fickian diffusion which is related with controlled swelling. [42,43].

## 3. Results and Discussion

### 3.1. Preparation and Characterization of Chitosan Microspheres

**Figure 3:**
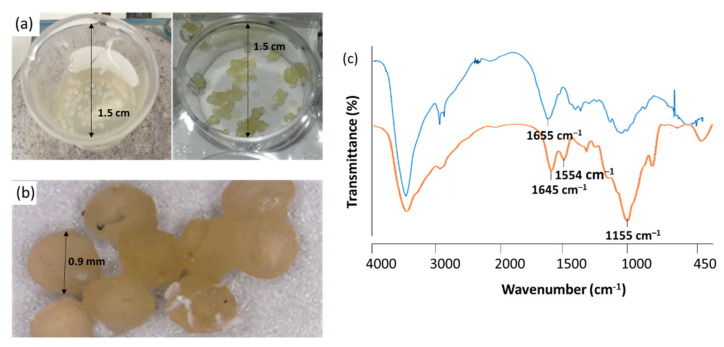
(**a**) Ch/TPP/Alg beads formation medium during Ch addition (left) and after precipitation (right); (**b**) microscopic view of microspheres sediment; (**c**) FTIR spectrum of Ch (blue) and Chitosan/TPP/Alg beads (orange).

Supramolecular chitosan beads were generated via dropwise addition of an aqueous Ch or Ch/AGA solution into either TPP or TPP/alginate solutions through a 20-gauge syringe needle, equilibrating the resulting mixtures (which successfully formed millimeter-scale spherical beads) at 25 °C for at least 15 min. Preliminary experiments were carried out in order to determinate the appropriate concentration range for chitosan, alginate and sodium tripolyphosphate, as well as other experimental conditions such as pH and polymers mass ratio. Modifications to the conditions were introduced along the optimization process to identify the best experimental conditions which were evaluated considering physical stability of microspheres suspensions (general aspect, formation of aggregates) and microspheres characteristics, such as size distribution. The obtained results indicated that the Ch/TPP microspheres are formed with better consistency when a 6:8 *w*/*w* TPP to chitosan mass ratio (*w*/*w*) has been employed, while in the case of Ch/ /TPP/Alg ones, a 4:8:6 *w*/*w*/*w* alginate to TPP to chitosan mass ratio (*w*/*w*/*w*) gave the best results. In both cases, the procedure developed provided spherical beads of homogeneous surface with no tendency to aggregate (Figure 3a,b). 

FTIR spectroscopy was employed to identify the functional groups of the prepared beads. The FTIR spectra of chitosan and Chitosan/TPP/Alg are shown in Figure 3c. In FTIR spectrum of Ch, the band at 1655 cm^–1^ is attributed to the –NH bending of the primary amine [44]. As can be observed in Figure 3c, this band disappears in the FTIR spectra of cross-linked chitosan, whereas two new peaks at 1645 cm^−1^ and 1554 cm^−1^ appear [45]. The disappearance of the band could be attributed to the complexation via electrolyte interactions between the negative ionic groups of TPP or Alg with Ch. The cross-linked Ch also showed a peak for P = O at 1155 cm^−1^ [46]. The adsorption of AGAs onto Ch beads did not show any significant additional peaks due to its similar functional groups that were overlapped in their peaks in the FTIR spectra of microspheres.

**Figure 4:**
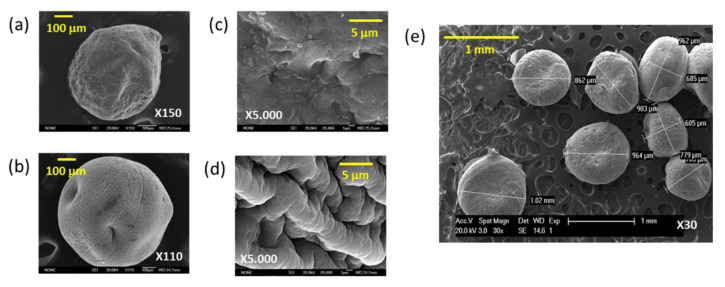
(**a**,**b**) Scanning electron microscope (SEM) image of Ch/TPP (**a**) and Ch/TPP/Alg beads (**b**); (**c**,**d**) Magnified view of Ch/TPP (**c**) and Ch/TPP/Alg beads (**d**); (**e**) Ch/TPP/Alg morphology.

The morphology and sizes of microspheres were analyzed by Scanning Electron Microscopy (SEM). Results show that the average diameter of the Ch/TPP beads is about 600 μm (Figure 4a), which is smaller than that obtained when Alg is introduced into the formulation, whose average diameter is about 900 μm (Figure 4b). Furthermore, in the SEM micrographs, qualitative topographical differences can be observed between particles. In particular, the Ch/TPP system shows a flatter surface (Figure 4c) than the Ch/TPP/Alg, whose surface is much more granular (Figure 4d). This topographic change could be due to the incorporation of Alg and the consequent formation of PECs complexes by interaction between Alg’s carboxylic groups and Ch’s amines [47]. In addition, as can be observed in Figure 4e, the bead shapes are spherical and uniform in size.

**Figure 5:**
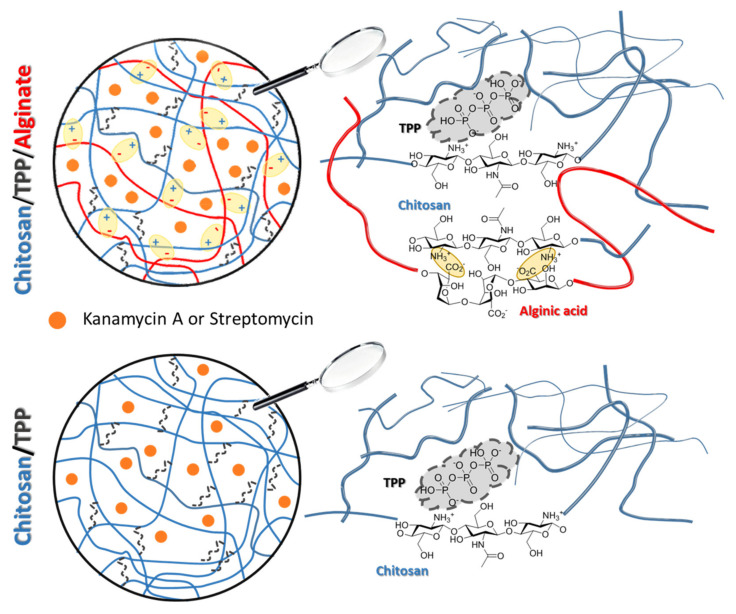
Graphical view of the Alg-coated chitosan/TPP and chitosan/TPP microspheres.

In Figure 5 a graphical representation of the chemical structure of microspheres is showed. In both cases, TPP is present in the bulk of the microspheres bridging between positive charges of the chitosan chains [48]. When Alg is added, this binds chitosan on the surface of the microspheres, although a certain degree of diffusion in the bulk is possible.

**Figure 6:**
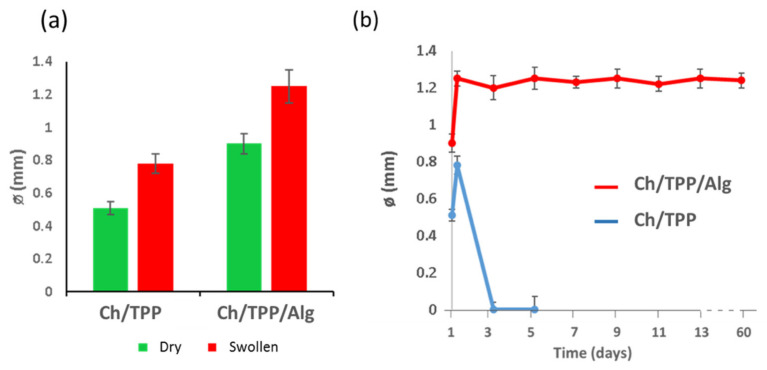
(**a**) Effect of microspheres composition on swelling after incubation in saline PBS (pH = 7.4) for 5 h. (**b**) Degradation of microspheres.

Swelling of the developed particles was studied under physiological conditions (saline PBS, pH = 7.4), and the results are represented in Figure 6a. Ch/TPP microspheres showed higher percentage of water uptake (≈52%) in comparison with Ch/TPP/Alg ones (≈38%). This reduction in the water absorption capacity could be explained on the basis that the additional coating with alginic acid of the microsphere would form a dense ionic cross-linking on the surface of the bead that would act as a diffusion barrier, thus decreasing its capacity for water absorption. This barrier would be formed as a result of ionic interactions between carboxylic groups of alginic acid and chitosan amines. For all microspheres, the faster water uptake was observed during the first two hours, reaching a stable value after 5 h, when equilibrium was attained. Finally, overall structural integrity of Ch/TPP/Alg microspheres was maintained in terms of form and size for at least two months, while Ch/TPP ones suffered further degradation after a few days (Figure 6b). Therefore, the first formulation was selected as the best formulation to use for all subsequent experimentation.

### 3.2. Antibiotic Efficiency

**Figure 7:**
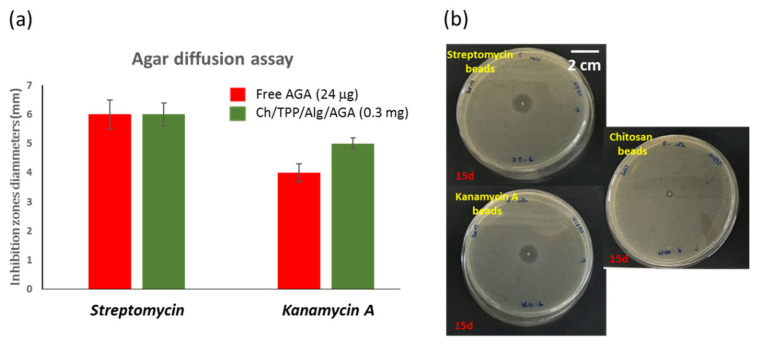
(**a**) Inhibition zone diameters (mm) of the free AGAs (red) and Ch/Alg/TPP/AGA (green) after 24 h. Error bars represent the standard errors. (**b**) Antimicrobial agar-diffusion assay of microspheres after 15 days.

The first objective of our study was to identify the antimicrobial properties of microspheres. In order to assess the antimicrobial activity of microspheres, the zones of bacterial growth inhibition were measured for the streptomycin and kanamycin loaded, in comparison with that of free antibiotics (Figure 7a). Streptomycin and kanamycin A are powerful antibiotics with MICs of 5 and 4 μg mL^−1^, respectively, for the *E. coli* BL21 (DE3) strain [49,50]. The agar diffusion results showed that, after 24 h, the AGA released from the beads was able to inhibit the bacterial growth of Gram-negative *E. coli* bacteria with a similar efficiency, or even better, in the case of kanamycin A, despite the amount of released-AGA, it should be significantly less than that of free-AGA. In addition, it has been observed that encapsulation of AGA prolonged its shelf life (at least 2 weeks). Thus, while free-AGAs showed strongest antibiotic activity for three days, the microspheres maintain this activity for a longer period (Figure 7b).

**Figure 8:**
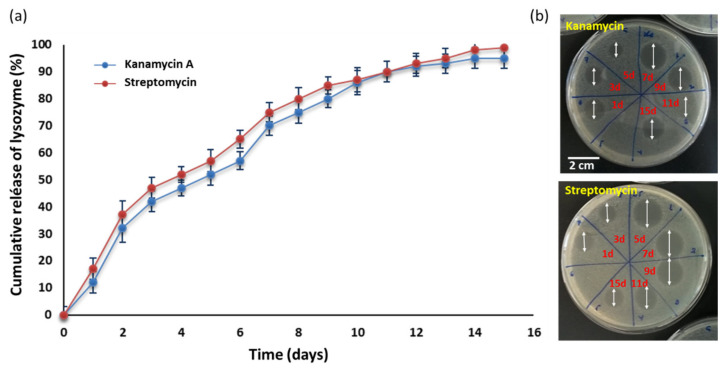
(**a**) In vitro release of kanamycin and streptomycin from the Ch/TPP/Alg microspheres. The mean cumulative concentration of antibiotic was expressed as a function of immersion time. (**b**) Examples of antibiotic release based on their antibacterial activity against *E. coli* BL21 (DE3) strain.

To go deeper into the antibiotic efficiency of microspheres, release kinetics of antibiotics from the Ch/TPP/Alg microspheres were measured (Figure 8a). AGAs absorb neither UV nor visible light. Hence, indirect analysis was performed to estimate the concentrations of the antibiotic, using aliquots antibiotic activity characterization by agar diffusion experiments (Figure 8b). The selection of this method has been based on the fact that the inhibition zone diameter increases proportionally with the antibiotic concentration [51]. 

The in vitro studies were performed in PBS (pH = 7.4) at 37 °C, and the obtained cumulative percentage of drug release was plotted against time to obtain the drug release profile. 

The relative faster antibiotic release during the first two days for both AGAs during the first three days (33.5% for kanamycin and 38.1% for streptomycin) when compared with the slower sustainable release during the successive days of the experiment could be due to an initial easily released AGA that is attached to the Alg-surface of microspheres. In fact, other authors have recorded a total burst of gentamicin, another antibiotic of the AGA-family, released from Alg-hyluronic acid microspheres during the first hours [52]. They proposed that this initial burst release may have also been due to the release of gentamicin molecules that were adsorbed on the surface of microspheres.

However, and despite the rapid initial release, if it is taken into account that the average drug loading efficiency was 8%, the total AGA in 0.3 mg of beads employed in the assay must be 24 μg, of which 3.8 μg of streptomycin and 4.3 μg of kanamycin A should be released in the first day of the experiment (see Figure 8a). According with these results, AGA-loaded beads prepared in this study have an increased antibacterial effect compared with the AGA solution again alone. This phenomenon could be due to a possible contribution of microspheres to the antibiotic activity. To analyze this possibility, we tested the antibacterial activities of Ch/TPP/Alg on *E. coli*. As shown in Figure 7b, Ch/TPP/Alg at an equal chitosan and alginate concentration to that in the loaded beads can inhibit the growth of *E. coli*. This indicates that, in addition to their role as carriers for antibiotics, microspheres contribute to the efficient antibacterial activity. These results are in agreement with those reported by other authors who found an increase in the inhibition ability of chitosan-alginate microspheres loaded with antibiotics in comparison with antibiotic alone, which confirms the antimicrobial potency of the microspheres [53,54,55]. Finally, to predict and correlate the release of AGAs from microspheres in vitro, results must be fit into a suitable mathematical model [41]. Among the different mechanisms known to control these processes, swelling, diffusion and erosion are the most accepted. In our study, we have used several conventional kinetic models, such as the zero-order, first-order, Higuchi and Hixon–Crowell ones. Table 1 shows the results of the kinetic modeling obtained for both antibiotics in our system.

**Figure 9:**
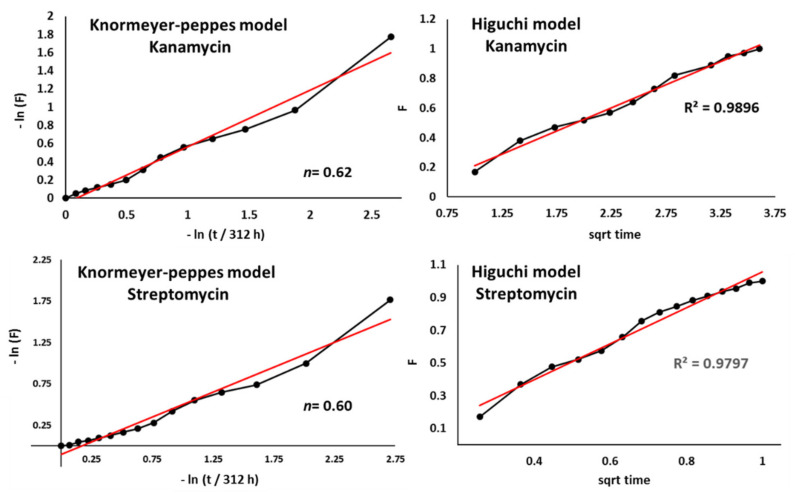
Drug release data fitted to Korsmeyer–Peppas and Higuchi models for kanamycin and streptomycin release (sqrt = square root of time).

Starting with the Korsmeyer–Peppas model, we obtained a release exponent value *n* of 0.62 for kanamycin and 0.60 for streptomycin, which is indicative of an anomalous release profile with a release mechanism controlled by both swelling and diffusion mechanisms (Figure 9). To further investigate whether diffusion or swelling was the governing mechanism of both antibiotics release out of the microspheres, other models have been applied (Table 1). 

Among all the analyzed models, the Higuchi model showed the highest coefficient of determination (R2), which indicates that a Fickian diffusion mechanism governs the release of AGAs from the microspheres (Figure 9). According to this model, the release of antibiotics appears to be a predominantly diffusion-controlled process, so that the liquid penetrates the beads, dissolving the antibiotics found inside.

These data are in concordance with a fast release at the initial stage due to the water wake-up of the microspheres, followed by a slower release during the successive days of the experiment. It should be noted that although the Higuchi model satisfactorily explains our drug delivery system, a more detailed mathematical analysis would be necessary to obtain more definitive conclusions.

Thus, our findings suggest that Ch/TPP/Alg microspheres can be proposed as efficient carriers for aminoglycoside antibiotics that permit the initial fast antibiotic release, which exceeds the minimal antibiotic therapeutic concentration (1 to 4 μg/mL) [56]. The present findings thus fit the initial need for high concentrations of antibiotic for infection treatments and avoid the sub-inhibitory antibiotic concentrations that can lead to a problematic resistance.

## 4. Conclusions

In this study, we report the synthesis and characterization of novel chitosan gel beads as a valuable platform for controlled release of the aminoglycosides streptomycin and kanamycin A. A double ionic co-crosslinking with TPP and alginate was used to prepare the chitosan-based networks of gel beads which were characterized in terms of their size, composition, and morphology. This particle formulation showed a sustained and controlled release of streptomycin and kanamycin based on a Fickian diffusion mechanism, which implies the solution of embedded antibiotics by liquid penetration inside the microsphere. Thus, during the first three days, a burst release was observed due to the fast water wake-up of the microspheres, whist in the next days, a slower diffusion mediated sustained release was observed for over 11 days. Moreover, these antibacterial particles were capable of effectively inhibiting *E. coli* strains’ growth. This behavior could be replicated also against other Gram-negative bacteria.

In sum, the novel antibacterial particles provide a means for increasing drug viability and stability while reducing the chances of drug accumulation into non-targeted tissues taking advantage of their sustained release properties. 

## Figures and Tables

**Table 1:** Kinetic release variables derived from mathematical models to describe AGA release from the microspheres.

Mathematical Model	Constant	Value	Constant	Value
	Kanamycin		Streptomycin	
Korsmeyer–Peppas	K	0.96	K	1.00
	R^2^	0.982	R^2^	0.970
	n	0.62	n	0.60
Zero-order	K_O_	0.83	K_O_	0.81
	R^2^	0.955	R^2^	0.918
First-order	K_1_	3.58	K_1_	4.20
	R^2^	0.937	R^2^	0.898
Higuchi	K_H_	0.31	K_H_	1.09
	R^2^	0.989	R^2^	0.979
Hixon−Crowell	K_S_	0.58	K_S_	0.38
	R^2^	0.951	R^2^	0.821

## Data Availability

Not applicable.
